# Isolated Ovarian Cyst Rupture Causing Hemoperitoneum After High-Energy Blunt Abdominal Trauma: A Case Report

**DOI:** 10.7759/cureus.102526

**Published:** 2026-01-28

**Authors:** Çağrı Akalın, Mümin Demir, Deha Denizhan Keskin

**Affiliations:** 1 General Surgery, Ordu Training and Research Hospital, Ordu, TUR; 2 Obstetrics and Gynaecology, Ordu Training and Research Hospital, Ordu, TUR

**Keywords:** blunt abdominal trauma, fertility preservation, hemoperitoneum, ovarian cyst rupture, pelvic fracture

## Abstract

Hemoperitoneum after high-energy blunt abdominal trauma is most commonly caused by solid organ injuries, particularly involving the liver and spleen, while isolated gynecological sources are rare and may be overlooked during initial evaluation. We report the case of a 23-year-old woman who presented after a fall from the fourth floor with hemodynamic instability and signs of an acute abdomen. Imaging revealed massive hemoperitoneum without evidence of solid organ injury, along with extensive pelvic fractures. Emergency exploratory laparotomy was performed due to persistent instability, and no general surgical source of bleeding was identified; instead, the sole cause of hemorrhage was a ruptured 12-cm ovarian cyst. Hemostasis was achieved through local hemostatic suturing while preserving the ovarian parenchyma. The postoperative course was uneventful. This case highlights that isolated ovarian cyst rupture, although rare, may represent the sole source of life-threatening hemoperitoneum following high-energy blunt abdominal trauma. In women of reproductive age, gynecological causes should be considered when no solid organ injury is identified, and exclusion of urological injury in the presence of pelvic fractures may facilitate focused and timely surgical management.

## Introduction

Blunt abdominal trauma (BAT) is a major cause of morbidity and mortality. It often involves injury to several organs. The liver and spleen are the most commonly injured and are the leading sources of traumatic hemoperitoneum [1]. Gynecological injuries are rare in trauma, accounting for less than 1% of cases, and usually coincide with other organ injuries [2].

Ovarian cyst rupture is a recognized gynecological emergency. It most often occurs spontaneously, often with functional cysts, such as corpus luteum cysts [3,4]. These cases are often self-limiting and managed conservatively if the patient is hemodynamically stable [5]. Rupture of an ovarian cyst due to high-energy BAT is exceedingly rare, especially as the sole cause of massive hemoperitoneum [6-8].

In women of reproductive age with traumatic hemoperitoneum, diagnosis often targets solid organ or major vascular injuries and may delay detection of gynecological bleeding, especially when imaging is unclear or findings are concealed by injuries such as pelvic fractures [2,9]. We present a rare case of isolated ovarian cyst rupture causing massive hemoperitoneum after high-energy BAT.

## Case presentation

A 23-year-old woman with no significant past medical history presented to the emergency department after falling from the fourth floor (about 12 meters). On arrival, she was conscious and oriented with a Glasgow Coma Scale score of 15. Her vital signs showed tachycardia (heart rate 120/min) and hypotension (blood pressure 95/60 mmHg). A physical examination revealed marked abdominal distension and generalized tenderness, particularly in the lower abdomen, along with guarding and rebound tenderness.

Laboratory results showed a hemoglobin of 7.3 g/dL, hematocrit of 23.8%, white blood cell count of 19,010/μL, platelet count of 216,000/μL, and an INR of 1.1. Serum β-human chorionic gonadotropin was negative. Focused assessment with sonography for trauma (FAST) revealed free intraperitoneal fluid in all quadrants.

**Figure 1:**
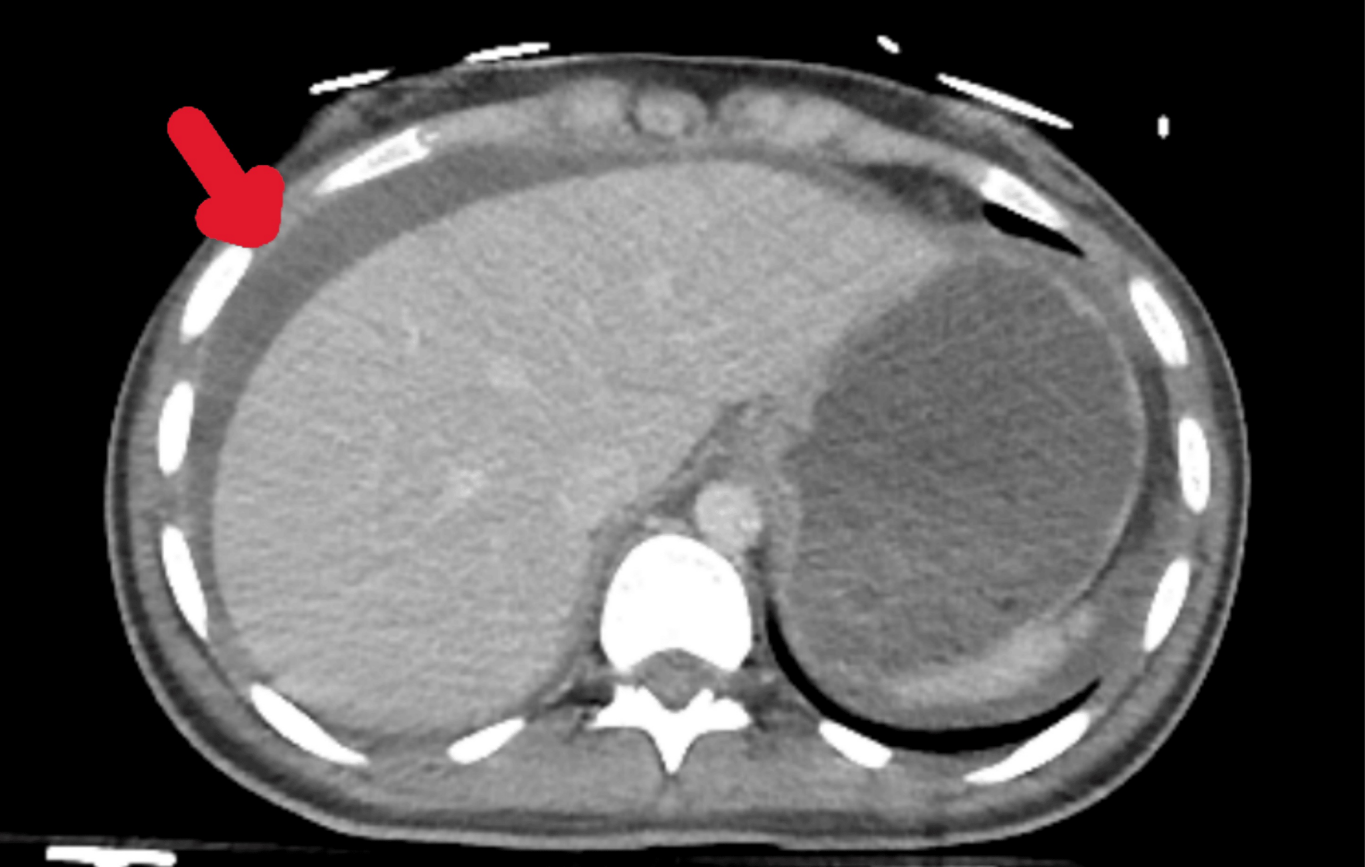
Computed tomography demonstrating diffuse hemoperitoneum (red arrow) without evidence of solid organ injury.

Contrast-enhanced computed tomography (CT) demonstrated massive hemoperitoneum without evidence of liver, spleen, bowel, or mesenteric injury (Figure 1). Additional findings included bilateral pulmonary contusions and multiple pelvic fractures involving the sacrum, both pubic rami, and the right acetabulum. Given the extent of pelvic fractures, preoperative retrograde cystography was performed to evaluate for bladder injury; however, no contrast extravasation was detected. Despite appropriate damage control resuscitation, including early balanced transfusion with 2 units of packed red blood cells and 1 unit of fresh frozen plasma along with restricted crystalloid administration (500 mL), the patient remained hemodynamically unstable. Therefore, an emergency exploratory laparotomy was performed.

**Figure 2:**
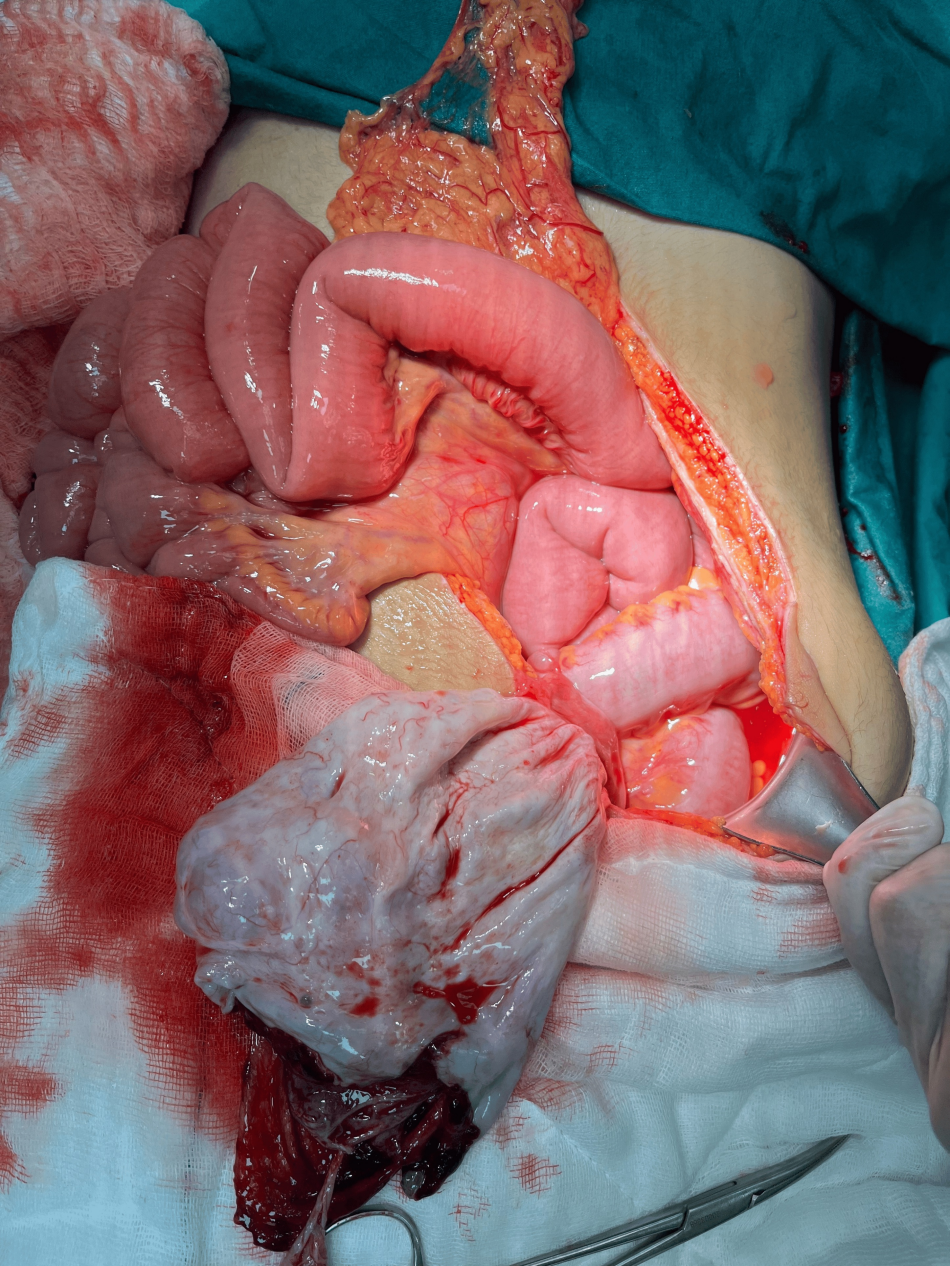
Intraoperative view demonstrating a ruptured left ovarian cyst, identified as the sole source of intraperitoneal hemorrhage.

Intraoperatively, approximately 1,200 mL of blood and clots were evacuated. Systematic exploration revealed no injury to the liver, spleen, bowel, mesentery, or bladder. The only bleeding source was a ruptured 12-cm left ovarian cyst, confirming adnexal hemorrhage (Figure 2). Local hemostasis of the ruptured ovarian cyst was achieved with figure-of-eight sutures (2-0 Vicryl), preserving the ovarian parenchyma without cyst excision. After completion of the emergency general surgery and gynecological procedures, orthopedic stabilization with external pelvic fixator application for pelvic ring stabilization was performed in a separate operative session once the patient’s hemodynamic condition had stabilized. Due to the severity of injuries, including bilateral pulmonary contusions and multiple fractures, the patient required 37 days in the intensive care unit. The patient was discharged home on postoperative day 48 with no complications.

## Discussion

Hemoperitoneum after BAT is usually due to solid organ injuries, especially the liver and spleen. These account for the majority of severe intra-abdominal bleeding in trauma patients [1]. By contrast, gynecological causes are rare, representing less than 1% of cases, and are often concealed by other injuries in polytrauma [2]. Isolated ovarian cyst rupture resulting in life-threatening hemoperitoneum following high-energy BAT represents an exceptionally rare clinical entity and poses a significant diagnostic challenge.

Ovarian cyst rupture typically occurs spontaneously, often with functional cysts like the corpus luteum, which are highly vascular and prone to bleeding [3,4]. Conservative management is usually successful in stable patients [5]. Traumatic rupture is very rare and almost always appears as isolated case reports [6-8]. This case shows that isolated ovarian cyst rupture can be the only source of massive hemoperitoneum after high-energy BAT.

Preoperative diagnosis of ovarian cyst rupture in trauma cases is difficult. CT is sensitive for detecting hemoperitoneum but may not show the bleeding source if there are no solid organ or major vascular injuries [9]. Diagnosis is even harder in the presence of pelvic fractures, which shift suspicion toward urological injuries like bladder rupture [10]. In this case, preoperative cystography excluded bladder injury, allowing focused surgical exploration and quick identification of the bleeding source.

Treatment of ruptured ovarian cysts depends on patient stability and the extent of hemorrhage. Conservative care is possible in stable patients. Surgery is needed if bleeding is ongoing or the patient is unstable [3,5]. Our patient required immediate laparotomy because she was unstable with a large-volume hemoperitoneum. Hemostasis was achieved through ovarian repair, not oophorectomy. This case shows that ovarian preservation is possible even with severe bleeding and should be attempted in reproductive-age women. Fertility preservation represents a critical consideration in the surgical management of reproductive-age women presenting with gynecological hemorrhage. Even in the setting of hemodynamic instability and large-volume hemoperitoneum, ovary-sparing procedures should be prioritized whenever feasible. Preservation of ovarian tissue not only maintains reproductive potential but also prevents the long-term endocrine consequences associated with oophorectomy. This case demonstrates that timely surgical intervention and meticulous hemostasis can allow ovarian preservation despite severe traumatic bleeding.

This case highlights important points. First, clinicians should consider gynecological bleeding in reproductive-age women with traumatic hemoperitoneum when no solid organ injury is identified. Second, imaging alone may not identify the bleeding source. Additional tests like cystography provide value in complex trauma. Third, early teamwork between surgery, gynecology, and trauma teams aids diagnosis, surgical planning, and fertility preservation. As a single case report, these findings cannot be generalized; however, they emphasize an important diagnostic consideration that may otherwise be overlooked in polytrauma settings.

## Conclusions

Isolated ovarian cyst rupture is rare but can cause life-threatening hemoperitoneum after high-energy BAT. Keeping a broad differential and systematically ruling out other bleeding sources helps prevent delay and allows for fertility-preserving surgery in reproductive-age women with traumatic hemoperitoneum.

## References

[1] Ashley JR, Burczak KW, Cotton BA, Clements TW (2024). Management of blunt abdominal trauma. Br J Surg.

[2] McWilliams GD, Hill MJ, Dietrich CS 3rd (2008). Gynecologic emergencies. Surg Clin North Am.

[3] Fiaschetti V, Ricci A, Scarano AL (2014). Hemoperitoneum from corpus luteal cyst rupture: a practical approach in emergency room. Case Rep Emerg Med.

[4] Roche O, Chavan N, Aquilina J, Rockall A (2012). Radiological appearances of gynaecological emergencies. Insights Imaging.

[5] Kim JH, Lee SM, Lee JH (2014). Successful conservative management of ruptured ovarian cysts with hemoperitoneum in healthy women. PLoS One.

[6] Kimbrell BJ, Emami C, Petrone P, Asensio JA (2007). Ruptured ovarian cystic teratoma secondary to blunt abdominal trauma: a very unusual case. J Trauma.

[7] Xaplanteri P, Zacharis N, Potsios C, Zacharis G (2020). Post-traumatic rupture of the right ovary and liver after blunt abdominal trauma: a case report. Int J Surg Case Rep.

[8] Yadav R, Sarkar M, Alam J, Bagaria D (2023). Posttraumatic corpus luteal cyst rupture: a diagnostic enigma for massive hemoperitoneum. Cureus.

[9] Lee MS, Moon MH, Woo H, Sung CK, Jeon HW, Lee TS (2017). Ruptured corpus luteal cyst: prediction of clinical outcomes with CT. Korean J Radiol.

[10] Yeung LL, McDonald AA, Como JJ (2019). Management of blunt force bladder injuries: a practice management guideline from the Eastern Association for the Surgery of Trauma. J Trauma Acute Care Surg.

